# Alignment differs between patellofemoral osteoarthritis cases and matched controls: An upright 3D MRI study

**DOI:** 10.1002/jor.24237

**Published:** 2019-03-01

**Authors:** Erin M. Macri, Agnes G. d'Entremont, Kay M. Crossley, Harvi F. Hart, Bruce B. Forster, David R. Wilson, Charles R. Ratzlaff, Charlie H. Goldsmith, Karim M. Khan

**Affiliations:** ^1^ Centre for Hip Health and Mobility, Department of Mechanical Engineering The University of British Columbia Vancouver British Columbia Canada; ^2^ La Trobe Sport and Exercise Medicine Research Centre, College of Science, Health and Engineering La Trobe University Melbourne Australia; ^3^ Department of Physical Therapy Western University London Ontario Canada; ^4^ Centre for Hip Health and Mobility, Department of Radiology The University of British Columbia Vancouver British Columbia Canada; ^5^ Centre for Hip Health and Mobility, Department of Orthopaedics The University of British Columbia Vancouver British Columbia Canada; ^6^ Arthritis Centre and College of Medicine University of Arizona Tucson Arizona; ^7^ Faculty of Health Sciences Simon Fraser University Burnaby British Columbia Canada; ^8^ Department of Occupational Science and Occupational Therapy The University of British Columbia Vancouver British Columbia Canada; ^9^ Centre for Hip Health and Mobility, Department of Family Practice The University of British Columbia Vancouver British Columbia Canada

**Keywords:** knee osteoarthritis, 3D alignment, upright MRI, patellofemoral joint, tibiofemoral joint

## Abstract

Patellofemoral (PF) osteoarthritis (OA) is a prevalent and clinically important knee OA subgroup. Malalignment may be an important risk factor for PF OA. However, little is known about alignment in PF OA, particularly in an upright, weightbearing environment. Using a vertically‐oriented open‐bore MR scanner, we evaluated 3D knee alignment in 15 PF OA cases and 15 individually matched asymptomatic controls. We imaged one knee per participant while they stood two‐legged at four flexion angles (0°, 15°, 30°, 45°), and also while they stood one‐legged at 30° knee flexion. We calculated 3D patellofemoral and tibiofemoral alignment. Using mixed effects models, four of the five patellofemoral measures differed by group. For key measures, PF OA patellae were 6.6° [95%CI 5.0, 8.2] more laterally tilted, 2.4 mm [1.3, 3.5] more laterally translated, and at least 3.7 mm [0.2, 7.2] more proximally translated compared to controls (more with knees flexed). Alignment did not differ between two‐legged stance and one‐legged stance in either group. Statement of Clinical Significance: Our study demonstrated significant and clinically relevant differences in alignment between PF OA cases and controls in upright standing and squatting positions. Our findings were similar to those in previous studies of PF OA using traditional MR scanners in supine positions, supporting the clinical usefulness of existing methods aimed at identifying individuals who may benefit from interventions designed to correct malalignment. © 2019 The Authors. *Journal of Orthopaedic Research*® Published by Wiley Periodicals, Inc. on behalf of the Orthopaedic Research Society. 9999:1–9, 2019.

Patellofemoral (PF) osteoarthritis (OA) is highly prevalent^1^ and is associated with substantial pain, reduced function, and lower quality of life.^2^, ^3^, ^4^, ^5^ Knee OA often begins at the patellofemoral joint, preceding multicompartment disease.^6^, ^7^, ^8^ An international consortium^9^ in 2017 posited that knee malalignment (both patellofemoral and tibiofemoral) may increase patellofemoral joint stress, leading to OA. Given that alignment may be modifiable (e.g., with physiotherapy techniques such as bracing or taping, or in rare cases, surgery),^10^, ^11^, ^12^ a better understanding of alignment in individuals with PF OA is in line with global research priorities.^13^

Knee malalignment is associated with prevalence and severity of PF OA‐related structural features (such as osteophytes, cartilage damage, bone marrow lesions).^14^ Specifically, the patella is generally positioned in more lateral tilt, lateral displacement, and proximal displacement compared to knees without PF OA‐related structural features.

In our 2016 systematic review,^14^ we identified four key gaps in the literature regarding alignment in PF OA. First, studies specifically recruiting individuals with PF OA to investigate these relationships were rare^11^, ^15^—most often, the target population was knee OA or tibiofemoral OA.^14^ Second, an asymptomatic non‐OA comparison group was included in just one study,^11^ rendering it challenging to determine effect sizes of malalignment in PF OA compared to asymptomatic knees. Third, in PF OA, knee alignment evaluated with magnetic resonance imaging (MRI) was exclusively measured with participants in a supine position.^14^ Pain is typically experienced by these individuals during upright, weightbearing tasks involving a flexed knee.^16^, ^17^ Thus, it is clinically important to investigate between‐group differences in upright positions to determine how alignment behaves in functional positions. In the few upright MRI studies of patellofemoral pain or instability, alignment is worse compared to controls.^18^, ^19^, ^20^ It is unknown if similar findings exist in upright, weightbearing positions in PF OA.

The final gap in the literature relates to the fact that in people with PF OA, a knee may exhibit greater malalignment during one‐legged stance. One‐legged stance increases the demand on the hip muscles, both in terms of strength (increased load) and motor control (narrower base of support), and increases patellofemoral joint load as body weight is transferred to a single limb. In a painful knee, this could invoke strategies to reduce patellofemoral joint reaction force that might result in further altering patellar alignment.^23^ No studies have compared alignment in two‐legged stance to one‐legged stance.

In this study, we aimed to evaluate upright 3D knee alignment to test the following hypotheses: (i) the patella would be more laterally displaced and tilted, and more proximally translated, in PF OA compared to matched controls and (ii) lateral displacement and tilt, and proximal translation, would be greater in one‐legged stance than in two‐legged stance.

## METHODS

### Study Design

For this cross‐sectional analytic study with individually matched controls (Level III evidence), we recruited a convenience sample of 30 individuals: 15 with symptomatic PF OA, and 15 matched asymptomatic controls. A sample size of 30 was set a priori based on the recommended guidelines for use with our chosen statistical approach, mixed effects modeling.^24^ The first 10 participants with PF OA and 10 controls (irrespective of matching) able to complete repeated measures (i.e., adequate time in the scanner) were included in our previously published study^25^ to establish reliability of the method of measurement. All 30 participants (15 matched pairs) were included in the present study. Participant recruitment took place from August 2014 until August 2016. Interested individuals contacted us after seeing our recruitment ads (targeted emails, newsletters, and posted notifications). All participants provided informed, written consent. Ethics approval was obtained from The University of British Columbia Clinical Research Ethics Board (certificate number H13–01993).

### Participants

Inclusion criteria for PF OA cases were: (i) age 40 years or older; (ii) peri‐ or retro‐patellar knee pain; (iii) pain aggravated by at least one activity that loads the patellofemoral joint (e.g., squatting, climbing stairs); (iv) pain rated at least 3/10 on a numeric pain rating scale during aggravating activities; (v) pain during aggravating activities present most days of the past month; and (vi) radiographic evidence of patellofemoral osteophytes rated at least Kellgren and Lawrence (KL) grade 1^26^, ^27^ on skyline or lateral view radiographs. Radiographs were reviewed by a musculoskeletal radiologist (BBF).

Exclusion criteria for PF OA cases were: (i) generalized knee pain; (ii) concomitant pain from hip, ankles, feet, or lumbar spine; (iii) recent knee injections (past 3 months); (iv) planned lower‐limb surgery in the following 6 months; (v) body mass index (BMI) of ≥35 kg/m^2^; (vi) knee or hip arthroplasty, osteotomy, reconstruction, meniscectomy, or fracture; (vii) history of major traumatic knee injury requiring non‐weightbearing for at least 24 h (e.g., fracture, dislocation, complete ligament rupture); (viii) physical inability to participate in testing; (ix) contraindications to MR imaging; (x) contraindications to radiation and did not already have recent radiographs (past year); (xi) tibiofemoral joint OA severity of KL grade 3 or 4^26^; (xii) worse radiographic OA severity at the tibiofemoral joint than the patellofemoral joint (i.e., tibiofemoral joint OA of KL grade 2 was eligible for inclusion provided the patellofemoral joint OA was at least as severe as the tibiofemoral joint); or (xiii) inability to understand written and spoken English.

Controls were matched with cases on five attributes: Age (within 5 years),^28^ sex,^28^ ethnicity,^29^ BMI (within 5 kg/m^2^),^28^, ^30^ and current physical activity level (low, moderate, or high based on the International Physical Activity Questionnaire‐Short, IPAQ‐S).^31^ Exclusion criteria included knee or other lower‐limb symptoms in the past year, plus the same criteria as for those with PF OA, with the exception of the criteria relating to radiographic OA, because controls did not undergo radiographic imaging for ethical reasons.

All participants completed the EuroQol 5 Dimension, 5 Level (EQ‐5D‐5L) health status measure.^32^ In addition, cases completed the Knee injury and Osteoarthritis Outcome Score (KOOS),^33^ including a sixth subscale, the KOOS Patellofemoral Subscale (KOOS‐PF).^34^

### Imaging

In PF OA cases, we scanned the painful (or more painful) knee. In controls, we selected and scanned the knee based on leg dominance of their matched case's scanned knee. We acquired MR images in two scanners to benefit from the advantages of each. Our method used a single high field strength image for each participant to provide the high resolution needed for assigning bony coordinate systems, and a low field strength vertically open‐bore scanner to rapidly capture alignment in various weightbearing positions.^25^, ^35^, ^36^

We acquired rapid images in a vertically oriented open‐bore 0.5T MRI scanner (ParaMed MROpen Genoa, Italy) with a commercial knee surface coil (see Table 1). Participants were scanned in upright (two‐legged) at four knee flexion angles (0°, 15°, 30°, 45°). One additional scan was obtained upright, standing one‐legged at 30° knee flexion on the imaged knee. In total, there were five different positions. Our protocol was designed to optimize visualization of bony outlines of the patella, tibia, and femur, while minimizing stand time to mitigate the risk of introducing movement artefact or unnecessary pain provocation for study participants.

**Table 1:** MRI Sequence Parameters

	3.0T Philips Achieva T1w TSE	0.5T Paramed Open GE
Repetition time (ms)	700	415
Echo time (ms)	10	10
Field of view (mm)	320	300
Acquisition matrix size (pixels)	512 × 460	160 × 128
Reconstructed matrix size (pixels)	512 × 512	256 × 256
Slice thickness (mm)	2.0	4.0
Gap thickness (mm)	0.0	0.4
Flip angle (°)	90	45
Total scan time (min)	∼16^*^*^	∼1

T1w TSE: T1‐weighted Turbo Spin Echo; GE: 3D gradient echo

^*^*^ Scan times are approximate on account of the size of the knee, with larger knees requiring slightly longer scan times.

**Figure 1:**
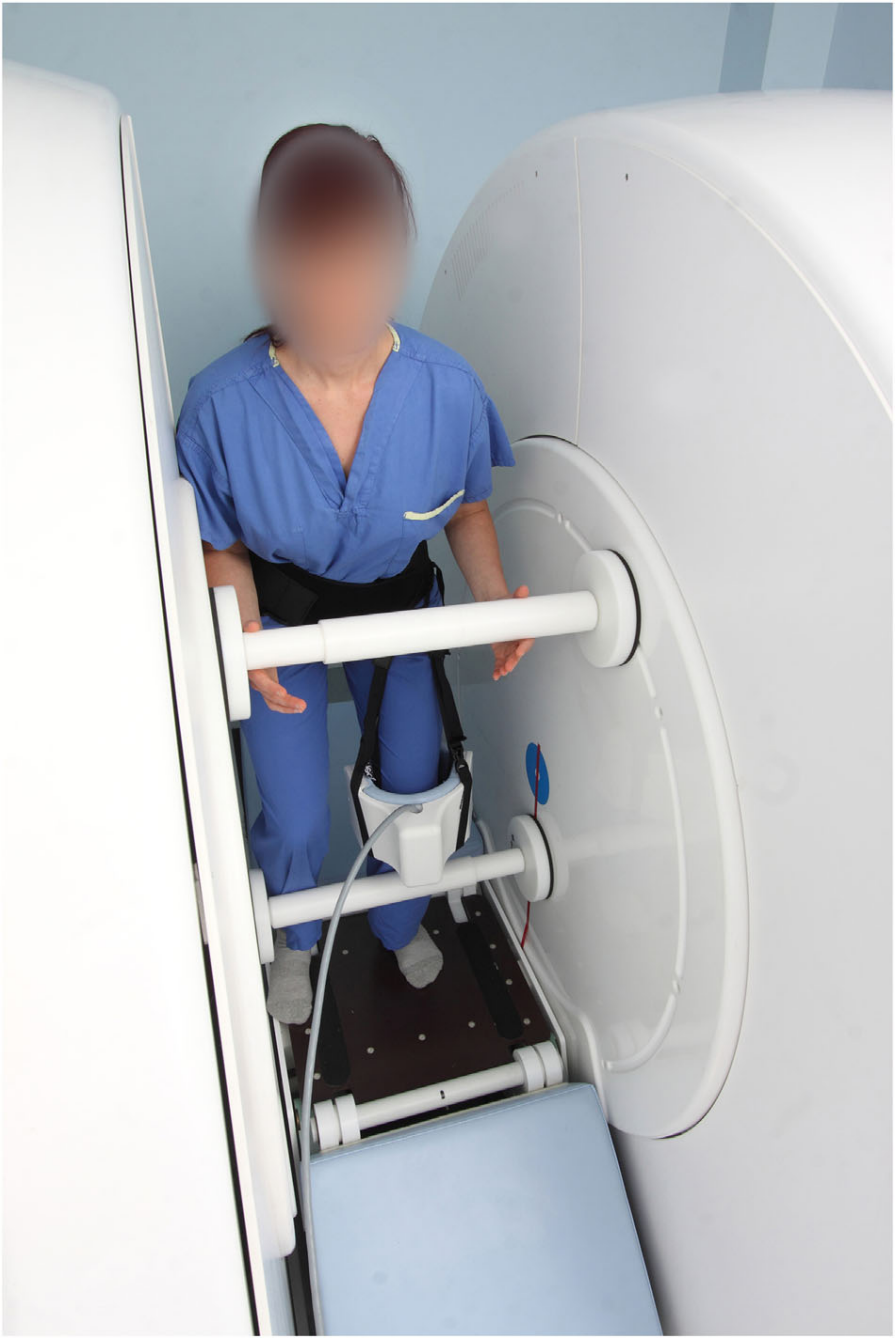
0.5T open‐bore scanner upright positioning. Participant stands on adjustable bench, a 12‐inch goniometer is used to achieve the correct knee flexion angle, then pressure bars are placed at the shins, buttocks, and at hand level for proprioceptive support, with feet positioned at 10° external rotation using a foot map (not shown). A plumb bob is used to ensure knees are not positioned anterior to the great toe. *Modified from Journal of Magnetic Resonance Imaging, Macri EM et al., “Patellofemoral and tibiofemoral alignment in a fully weight‐bearing upright MR: Implementation and repeatability” Published Online First: Doi:10.1002/jmri.25823, 2017, with permission from John Wiley and Sons*.

For standing images, participants stood two‐legged in the scanner (Figure 1).^25^ A 12‐inch goniometer was used to estimate knee flexion angle, and consistent methods were used to maintain position during image acquisition, including the placement of pressure bars intended to aid participants in achieving stillness during image acquisition (participants were instructed not put weight through the bars).^25^

We acquired high‐resolution sagittal images in a traditional closed‐bore 3T MRI scanner (Philips Achieva Best, NL) with a dual SENSE Flex‐M coil configuration (see Table 1).^25^ The large field‐of‐view, high resolution image produced was used to create participant‐specific 3D bone surface models (patella, femur, tibia), with coordinate systems attached, for the subsequent registration and bony alignment calculation processes.

### Alignment

Post‐image processing methods for calculating 3D alignment have been previously reported in detail.^25^, ^35^, ^36^ Briefly, we manually segmented bony outlines of the femur, patella, and tibia of the single high resolution (3T) image to create participant‐specific anatomical surface models.^35^ We then manually assigned orthogonal coordinate axes to each bone model using the high‐resolution MR images and Grood and Suntay's joint coordinate system methods.^36^, ^37^ Anatomical landmarks used to define axes were: Patella—(i) most lateral point of the mid‐patella; (ii) most posterior point of the central median ridge of the mid‐patella; (iii) most superior; and (iv) inferior points of the mid‐patella; femur—(i) most posterior points of the lateral and (ii) medial femoral condyles, (iii) intercondylar notch, and (iv) two proximal points in centre of femoral shaft (to define axis); tibia—(i) most posterior point of the lateral and (ii) medial proximal tibial condyles, (iii) medial tibial eminence, and (iv) two distal points in centre of tibial shaft (to define axis).

**Figure 2:**
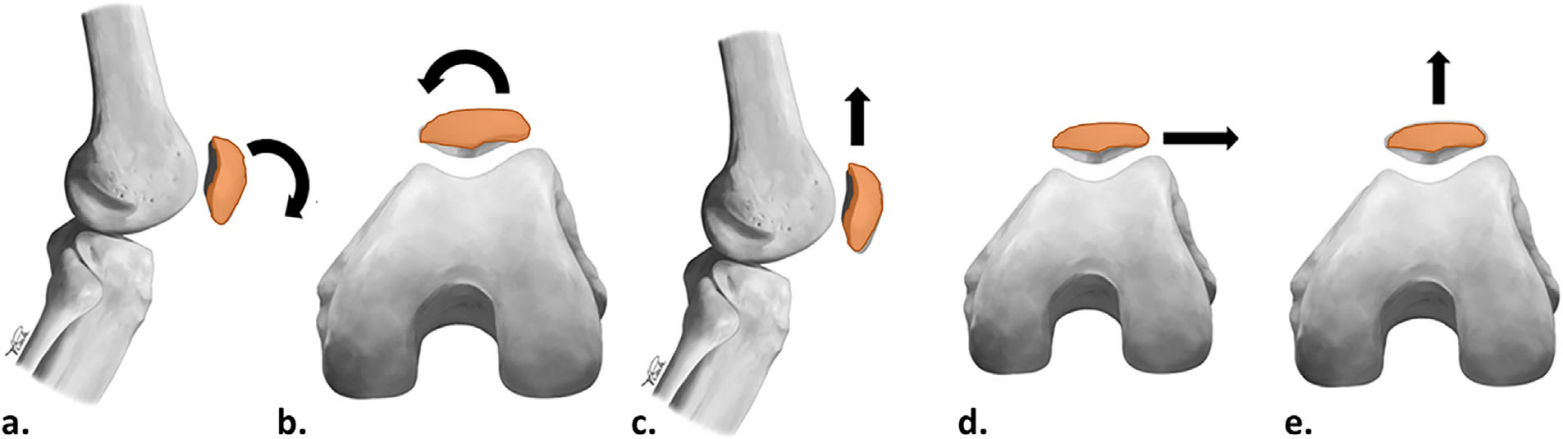
Patellar alignment: (A) flexion, (b) medial tilt, (c) proximal translation, (d) lateral translation, (e) anterior translation. Arrows show direction of increasing value for each alignment variable, and all values represent the position of the patella relative to the femur during static image acquisition. *Illustrations by Vicky Earle, modified from Journal of Magnetic Resonance Imaging, Macri EM et al., “Patellofemoral and tibiofemoral alignment in a fully weight‐bearing upright MR: Implementation and repeatability” Published Online First: Doi:10.1002/jmri.25823, 2017, with permission from John Wiley and Sons*.

Using the rapid, lower resolution images obtained in the 0.5T vertically open bore scanner, we manually segmented bony surface outlines for the femur, patella, and tibia. We then registered (shape‐matched) the high‐resolution anatomic surface models (with bone coordinate system attached) to each of the lower‐resolution bony outlines using an iterative closest points algorithm.^25^, ^35^, ^36^ Next, we calculated alignment of the patella relative to the femur (flexion, medial tilt, and proximal, lateral, and anterior translation) using the assigned joint coordinate system^37^ (see Figure 2 for specific alignment labeling conventions). We also calculated alignment of the tibia relative to the femur (see supplementary materials Figure S1).

The advantage of this method is that the bone coordinate systems are only applied once, using the high‐resolution 3T images. The participant‐specific models created from the 3T images, with coordinate systems attached, are then registered to each of the rapid images taken in the 0.5T scanner, eliminating the need to find the same landmarks repeatedly across multiple images and positions and thus reducing error.

Previously, using a cadaver model in a 1.5T scanner, overall error using these methods was determined to be ≤1.75° for rotations and ≤0.88 mm for translations, compared to a criterion measure (roentgen stereophotogrammetric analyses).^36^ Using living participants in a 1.5T scanner, mean registration error was reported to be ≤0.44° for rotations, and ≤0.30 mm for translations, and test‐retest (“intrasubject”) variability was ≤1.40° for rotations and ≤0.81 mm for translations.^35^ While these estimates were obtained with images obtained from a 1.5T scanner, we assessed repeatability in the current protocol (using a 3T and 0.5T scanner and image sequences with similar resolution) and standard error of measure values were <2° rotation and <0.9 mm translation,^25^ suggesting comparable error in this upright environment.

### Statistical Analyses

We used two statistical approaches to evaluate alignment outcomes as a function of group (PF OA or control) and knee flexion angle: Mixed effects models, and the difference method.

#### Inferential Statistics: Mixed Effects Models

We constructed mixed effects models^24^, ^38^ to evaluate differences between PF OA cases and controls across all angles and positions in a single model, with one model for each alignment variable (one‐legged stance was not included in these models). We treated group as a fixed effect, and, for the random portion of the model, we fit a random intercept and random slope for knee flexion angle (as a continuous variable based on the post‐image processing values) at the case‐control pair level. We considered square terms, centered on the grand mean, for tibiofemoral knee flexion angle (in case associations were curvilinear) and also considered all possible interaction terms for inclusion into the model.

We evaluated one‐legged stance at 30° knee flexion by comparing this position to standing two‐legged at 30° knee flexion. We created separate models for these analyses using the same methods.

#### Inferential Statistics: Difference Method

We calculated mean (SD) alignment differences between groups (PF OA cases minus controls) at all four angles of knee flexion. We also calculated Cohen's d standardized effect sizes to assist with clinical interpretation.^39^ Acknowledging that cut‐points are arbitrary, we defined a small effect size as 0.01 ≤ |d| ≤ 0.49, medium as 0.50 ≤ |d| ≤ 0.79, and large as |d| ≥ 0.8.^39^

All statistical analyses were completed using Stata 13 (StataCorp, TX). Statistical significance was defined as *p* ≤ 0.05, with no adjustments for multiple testing.

## RESULTS

**Figure 3:**
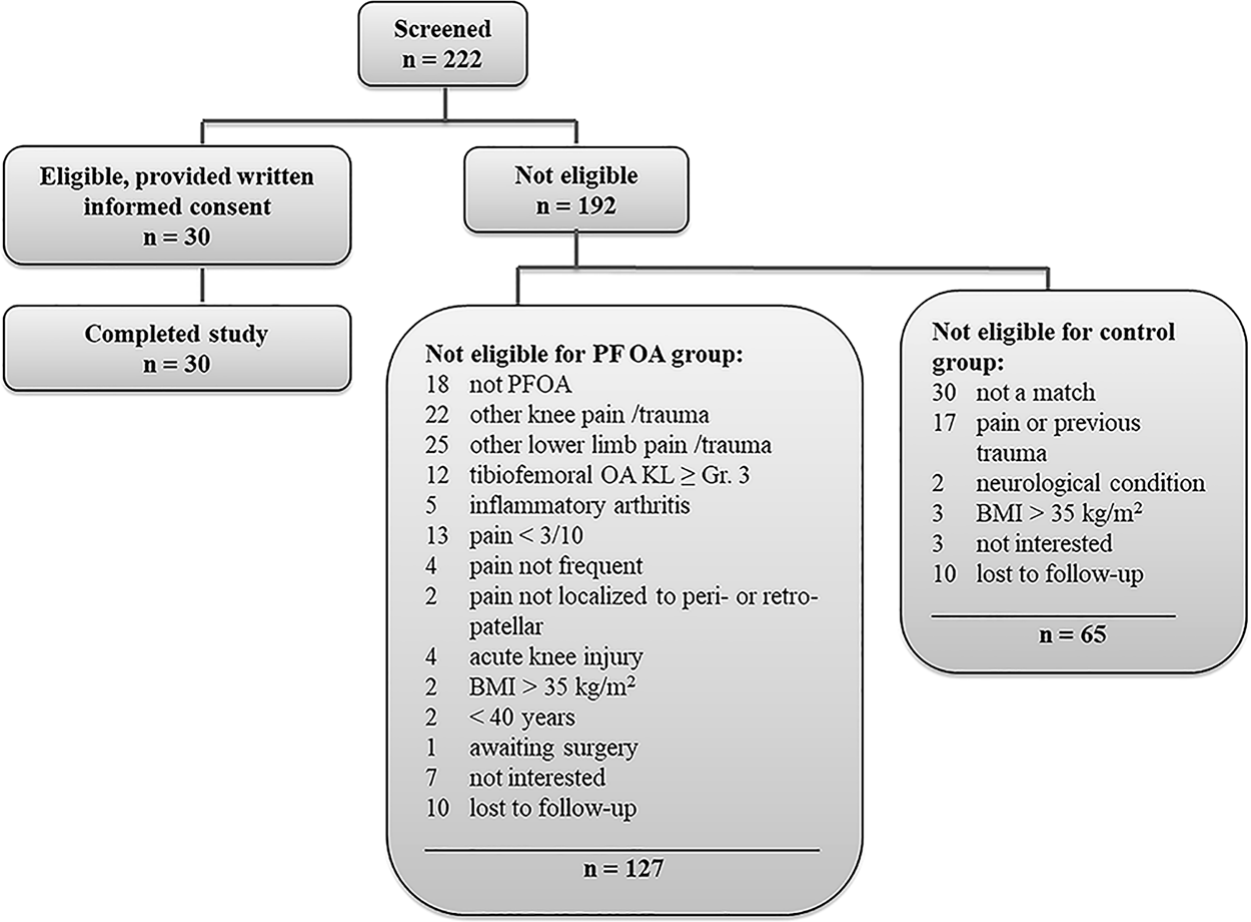
Flow chart for participant screening.

We screened 222 individuals until we completed recruitment of 30 participants (see Figure 3 for screening flow chart). The full sample (*n* = 30) had a mean (SD) age of 56 (7) years and BMI of 23.5 (3.7) kg/m^2^, and comprised 24 women (80%) (see Table 2). Twelve pairs (24 participants) were of European ethnicity, three pairs were of Chinese ethnicity, and one pair was of Indian/mixed Indian ethnicity. Nine pairs (18 participants) reported participating in moderate physical activity; four pairs reported high physical activity; and two pairs reported low physical activity. We report patellofemoral alignment below − for tibiofemoral alignment results, see supplementary materials (Table S1 and S2, Figure S2).

**Table 2:** Participant Characteristics (*n* = 30)

	PF OA (*n* = 15)	Controls (*n* = 15)
Age, years	56 (8)	56 (7)
BMI, kg/m^2^	23.5 (4.1)	23.5 (3.4)
Women, *n* (%)	12 (80%)	12 (80%)
EQ‐5D‐5L		
Index	0.866 (0.066)	0.939 (0.027)
VAS	85.0 (8.7) [70,100]	92.7 (6.4) [80,100]
KOOS		
Symptoms	68.8 (16.7) [32.1, 89.3]	‐
Pain	71.5 (11.6) [50.0, 86.1]	‐
Activities of daily living	82.9 (12.6) [54.2, 100.0]	‐
Sport and recreation	46.0 (28.1) [10.0, 95.0]	‐
Quality of life	48.3 (17.9) [18.8, 68.8]	‐
Patellofemoral	56.8 (17.9) [18.2, 77.3]	‐

BMI: body mass index; EQ‐5D‐5L: EuroQol Health Status Measure‐5 dimension‐5‐likert: Index provides a Canadian‐specific adjusted score combining all 5 dimensions (scores from zero [dead] to 1.000 [perfect health]), and VAS (visual analogue scale) is a single overall self‐reported evaluation that varies from zero (dead) to 100 (perfect health); KOOS: Knee injury and Osteoarthritis Outcome Score (varies from zero, maximum problems, to 100, no problems)

During data collection, we discovered that two participants had conditions with potential to influence study outcomes (one participant reported an asymptomatic neurologic disease, and one reported spinal scoliosis). We therefore located these two participants on all exploratory scatter plots, and in any measures where their values were positioned on the periphery of overall sample values (even if they were not frank influential observations) we conducted sensitivity analyses with those participants removed. The results of these analyses revealed that effect sizes were either no different or larger with the participants removed. We therefore report the more conservative results here for the full sample without removal of these two participants.

**Figure 4:**
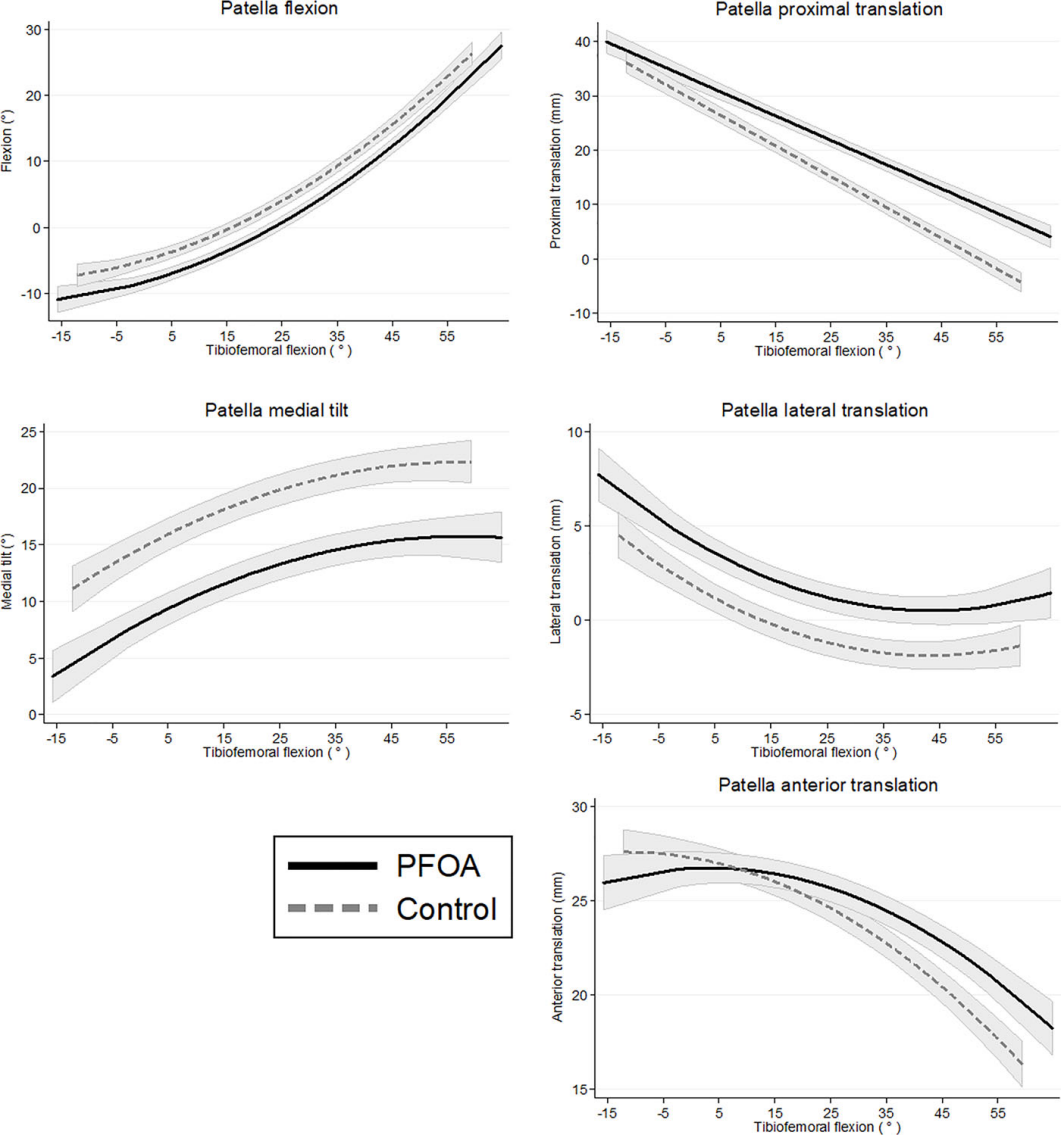
Fitted 3D patellar alignment results for PF OA cases (solid black) and controls (dashed grey) over a variety of tibiofemoral flexion angles. Gray shading represents one standard deviation above and below the group means. All values represent the static position of the patella relative to the femur.

In mixed effects models, PF OA cases had 3° less patellar flexion than controls through all knee flexion angles evaluated (Table 3, Figure 4). For patellar tilt, cases were 7° less medially (i.e., more laterally) tilted than controls through all knee flexion angles evaluated. PF OA cases had more proximally positioned patellae than controls, with larger differences at higher angles of knee flexion. The cases were approximately 2 mm more laterally translated than controls through all knee flexion angles. The cases were more anteriorly translated than controls when knees were flexed, with larger differences at higher angles of knee flexion.

**Table 3:** 3D Alignment, Mixed Effects Models: Model Coefficients ($\hat{\beta }$) with 95% Confidence Intervals (CI) and p‐values

Alignment	$\hat{\beta }$	95%CI	*p*
Patellar flexion (°)			
PF OA	−3.28	−4.80, −1.76	**<0.001**
TFJ flexion	0.49	0.44, 0.54	**<0.001**
TFJ flexion^2^	0.005	0.003, 0.007	**<0.001**
Patellar medial tilt (°)			
PF OA	−6.58	−8.19, −4.98	**<0.001**
TFJ flexion	0.15	0.10, 0.20	**<0.001**
TFJ flexion^2^	−0.002	‐0.005, 0.000	0.05
Patellar proximal translation (mm)			
PF OA	3.70	0.19, 7.21	**0.04**
TFJ flexion	−0.56	−0.64, −0.49	**<0.001**
PF OA*TFJ flexion	0.12	0.01, 0.23	**0.04**
Patella lateral translation (mm)			
PF OA	2.38	1.29, 3.47	**<0.001**
TFJ flexion	−0.07	−0.10, −0.04	**<0.001**
TFJ flexion^2^	0.002	0.001, 0.004	**0.008**
Patella anterior translation (mm)			
PF OA	−0.57	−2.19, 1.05	0.49
TFJ flexion	−0.17	−0.21, −0.13	**<0.001**
TFJ flexion^2^	−0.002	−0.004, −0.001	**0.001**
PF OA*TFJ flexion	0.07	0.01, 0.12	**0.01**

PF OA represents difference of PF OA cases minus matched controls; TFJ flexion = tibiofemoral joint flexion angle; TFJ flexion^2^ square term (centralized on the mean); * denotes interaction terms. Bold indicates *p* < 0.05.

Using the difference method, effect sizes were consistently moderate to large in patellar tilt (PF OA cases more laterally tilted than controls at all angles), moderate in patellar proximal translation (cases more proximal), and moderate in patellar lateral translation in full extension (cases more laterally displaced) (see Table 4).

**Table 4:** Mean (SD) Between‐group Difference in Alignment of Matched Pairs in Standing Two‐legged at Four Knee Flexion Angles

	Standing, two‐legged							
Patellofemoral joint	0° flexion	d	15°	d	30°	d	45°	d
Flexion (°)	−1.7 (7.6)	−0.2	−3.0 (6.7)	−0.4	−2.9 (8.2)	−0.4	−2.3 (10.3)	−0.2
Medial tilt (°)	−5.9 (9.1)	**−0.6**	−7.2 (9.2)	**−0.8**	−7.4 (7.7)	**−1.0**	−4.6 (5.6)	**−0.8**
Proximal translation (mm)	5.8 (12.5)	**0.5**	4.9 (11.3)	0.4	6.4 (12.2)	**0.5**	6.7 (10.4)	**0.6**
Lateral translation (mm)	2.4 (5.1)	**0.5**	2.5 (6.0)	0.4	2.4 (5.8)	0.4	1.7 (5.1)	0.3
Anterior translation (mm)	−0.1 (3.7)	0.0	0.3 (3.9)	0.1	1.3 (4.9)	0.3	2.5 (5.9)	0.4

All values report values as PF OA cases minus control, plus Cohen's d.

Bold indicates Cohen's |d| ≥ 0.5

### One‐Legged Squat

When standing one‐legged at 30° knee flexion, compared to standing two‐legged at the same angle, patellar tilt differed significantly within the case group, however the amount of change was small (0.9° more medial tilt) (Table 5).

**Table 5:** Within‐group difference in alignment, one‐legged minus two‐legged, standing at 30° flexion

	Patellofemoral OA (*n* = 15)			Control (*n* = 15)		
Patellofemoral joint	$\hat{\beta }$	95%CI	*p*	$\hat{\beta }$	95%CI	*p*
Flexion (°)	−1.01	−2.48, 0.46	0.18	−1.40	−2.66, −0.14	**0.029**
Medial tilt (°)	0.85	0.10, 1.61	**0.027**	‐0.20	‐1.43, 1.02	0.744
Proximal translation (mm)	0.66	‐0.37, 1.69	0.208	0.60	−0.09, 1.29	0.090
Lateral translation (mm)	−0.16	−0.72, 0.39	0.563	0.10	−0.43, 0.64	0.706
Anterior translation (mm)	−0.17	−0.74, 0.40	0.550	‐0.48	−0.88, −0.07	**0.021**

Mixed effects models.

Bold indicates *p* < 0.05.

## DISCUSSION

The results of this study support our hypotheses that the patella was more laterally displaced and tilted, and more proximally translated, in PF OA compared to controls. Alignment generally did not differ when standing one‐legged compared to standing two‐legged at 30° knee flexion.

Our findings extend those of alignment studies in other patellofemoral pathologies such as patellofemoral pain or instability, and indicate that alignment in PF OA may be similar to that seen in these other populations.^18^, ^19^, ^21^, ^22^ In studies of patellofemoral pain or instability, under various conditions in terms of position and loading and across different knee flexion angles, lateral translation, lateral tilt, and proximal translation were greater compared to controls, with larger differences often seen in full extension.^19^, ^20^, ^40^, ^41^ Our results were similar, but did not show larger between‐group differences in any measure in full extension. Our physiologically upright positioning, static imaging protocol, and 3D alignment measurement methods may in part explain our different results. However, it may also be that alignment in PF OA differs fundamentally compared to patellofemoral pain or instability, particularly when knees are fully extended.

Our study results did not support the hypothesis that malalignment would be greater when standing one‐legged compared to standing two‐legged at 30° knee flexion. It is possible that offering assistive devices for balance (pressure bars) reduced the challenge of standing on one leg, even though participants were instructed to not put weight through the bars. Alternatively, the additional load and complexity of this task was not sufficient to influence alignment. It may be that more difficult tasks such as jumping are required to increase the task load sufficiently to elicit changes in alignment.^42^, ^43^ Our results do not justify acquiring images in standing one‐legged, however this has not yet been evaluated at deeper angles of knee flexion, or with participants self‐selecting their position. Clinically, one‐legged stance may still be important—dynamic valgus is common with knee pathology, particularly with more demanding functional tasks.^44^, ^45^

Our study results suggest that alignment in upright positions in PF OA may be similar to those reported previously using traditional closed‐bore scanners.^14^ Thus, traditional closed‐bore scanners remain clinically useful in the ongoing evaluation of alignment in PF OA in comparison to healthy controls. Studies are needed to determine whether specific positions, loading conditions, knee flexion angles, or MR sequences are better for detecting alignment values with the greatest diagnostic accuracy for patellofemoral pain or OA.

### Limitations

This study is cross‐sectional and we cannot draw conclusions regarding causation between alignment and PF OA. In addition, there is debate as to the best criteria for defining and diagnosing PF OA. Including participants with doubtful osteophytes in our study resulted in including three participants who may be better described as having patellofemoral pain. However, doubtful osteophytes commonly progress to definite osteophytes,^28^ and we aimed to target early PF OA. All three participants with doubtful osteophytes met the National Institutes for Health and Care Excellence (NICE) diagnostic guidelines for knee OA.^46^

Another limitation is that we acquired images with knees positioned statically. Alignment differs between dynamic and static MR sequences in supine positions. It is not known to what extent this occurs in upright positions.^47^ However, dynamic MR scans in upright movements are typically limited to capturing a single slice image, and we chose a static method where we could evaluate the entire knee and use this to simultaneously calculate alignment in 3D.

Our study had a relatively small sample size. When using mixed methods modeling, a sample size of 10 per independent variable is considered adequate.^24^ Our results showed both statistically significant and clinically relevant effect sizes (based on standardized effect sizes). Previously established smallest detectible differences (SDC_95_) for comparing two measures are: Patellar flexion 3.2°, medial tilt 4.4°, proximal translation 2.4 mm, lateral translation 1.3 mm, and anterior translation 1.4 mm.^26^ Key differences in the present study exceeded SDC_95_ in all patellar alignment measures except anterior translation. Patterns of alignment were also biologically plausible and similar to previously published studies of patellofemoral disease. Study design factors such as sample size, sample selection, inherent methodological error, and multiple testing raise the possibility of Type I and Type II error.

Accuracy of our methods was previously established using a 1.5T MR scanner exclusively.^36^ However, we did not explicitly evaluate the accuracy (i.e., compared to the criterion roentgen stereophotogrammetric analyses) of our alignment values obtained using a 3T scanner (high resolution images, creation of participant‐specific anatomical surface models and assignment of bone coordinate systems) and 0.5T scanner (low resolution images, creation of bony surface outlines that were registered to the high resolution images) together.

Finally, more research is needed to support the clinical understanding of patellar alignment. For example, there is substantial overlap in patellofemoral alignment measures in those with and without patellofemoral OA or pain, and the risk of having OA or pain rises in a graded manner with increasing alignment values—there is no biologically obvious cut‐point for defining “malalignment”.^48^ Moreover, the minimal clinically important difference (MCID) has not yet been established for patellofemoral alignment measures. Thus, while we determined the results of the present study to be clinically relevant based on standardized effect sizes (Cohen's d), it is not known whether these values or differences exceed thresholds determined to be either malaligned or clinically important.

## CONCLUSIONS

We found that knees with PF OA had greater lateral translation, lateral tilt, and proximal translation compared to matched asymptomatic controls. Our study of participants with PF OA complements studies of participants with patellofemoral pain and instability and contributes to the growing body of literature that posits patellofemoral pain and PF OA may lie along a disease continuum.^49^ Future studies investigating interventions for PF OA should consider including mechanistic outcomes such as alignment (in addition to typical pain and function outcomes) to gain insight as to which interventions are most effective for addressing PF OA related malalignment.

## AUTHORS’ CONTRIBUTION

EMM, AGd, KMC, HFH, BBF, DRW, CRR, KMK contributed to study design and methodology; EMM, HFH, CHG, AGd, KMC contributed to acquisition, analysis, or interpretation of data; all authors contributed to drafting the paper or revising it critically; and all authors read and approved the final submitted manuscript.

## Supporting information

Additional supporting information may be found in the online version of this article.

Supporting Data S1.
