# Supplementary material for: Alignment differs between patellofemoral osteoarthritis cases and matched controls: An upright 3D MRI study
Source: J Orthop Res. 2019 Mar 1;37(3):640–8. doi: 10.1002/jor.24237 (PMC6593798; doi:10.1002/jor.24237)
Supplement: Supplementary file 1 — Supporting Data S1. [file JOR-37-640-s001.docx]

# Supplementary file 1. Tibiofemoral alignment methods and results


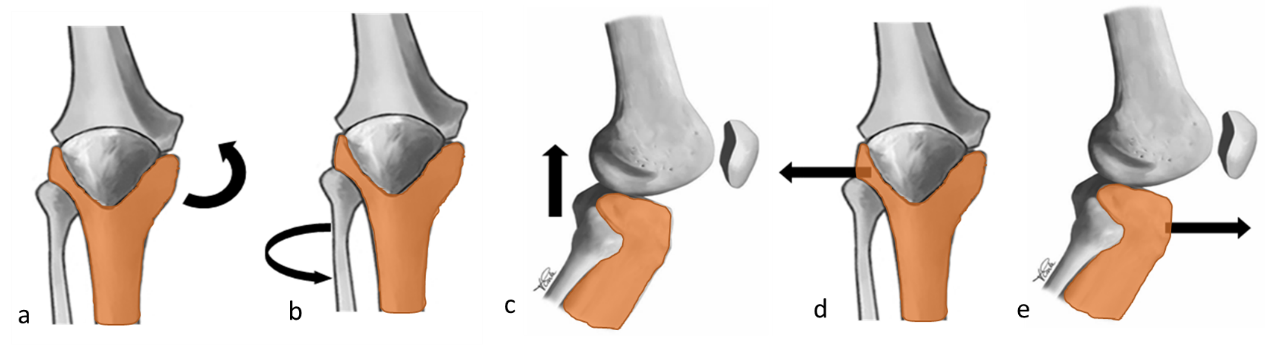


FIGURE S1. Tibial alignment: a. adduction; b. internal rotation; c. proximal translation; d. lateral translation; e. anterior translation. Arrows show direction of increasing value for each alignment parameter, and all values represent the position of the patella relative to the femur during static image acquisition. Illustrations by Vicky Earle. *Modified from Journal of Magnetic Resonance Imaging, Macri EM et al., “Patellofemoral and tibiofemoral alignment in a fully weight-bearing upright MR: Implementation and repeatability” Published Online First: doi:10.1002/jmri.25823, 2017, with permission from John Wiley and Sons.*

TABLE S1. Tibiofemoral alignment, mixed effects models: model coefficients ($\hat{\boldsymbol{\beta}}$) with 95% confidence intervals (CI) and p-values.

| **Tibiofemoral alignment** | $\hat{\boldsymbol{\beta}}$ | **95% CI** | **p** |
| --- | --- | --- | --- |
| **Tibial adduction** |  |  |  |
| PF OA | -0.18 | -1.07, 0.70 | 0.69 |
| TFJ flexion | 0.02 | -0.004, 0.05 | 0.10 |
| **Tibial internal rotation** |  |  |  |
| PF OA | -2.88 | -4.12, -1.63 | **< 0.001** |
| TFJ flexion | 0.25 | 0.20, 0.30 | **< 0.001** |
| TFJ flexion ^2^ | -0.003 | -0.005, -0.002 | **< 0.001** |
| **Tibial proximal translation** |  |  |  |
| PF OA | 0.75 | 0.24, 1.26 | **0.004** |
| TFJ flexion | 0.07 | 0.05, 0.09 | **< 0.001** |
| **Tibial lateral translation** |  |  |  |
| PF OA | 0.22 | -0.33, 0.77 | 0.43 |
| TFJ flexion | -0.05 | -0.06, -0.03 | **< 0.001** |
| **Tibial anterior translation** |  |  |  |
| PF OA | 2.12 | 1.08, 3.16 | **< 0.001** |
| TFJ flexion | -0.21 | -0.24, -0.18 | **< 0.001** |

*PF OA represents difference of PF OA cases minus matched controls; TFJ flexion = tibiofemoral joint flexion angle; TFJ flexion^2^ square term (centralized on the mean); Position represents difference of standing minus supine. * denotes interaction terms. Bold indicates p < 0.05.*


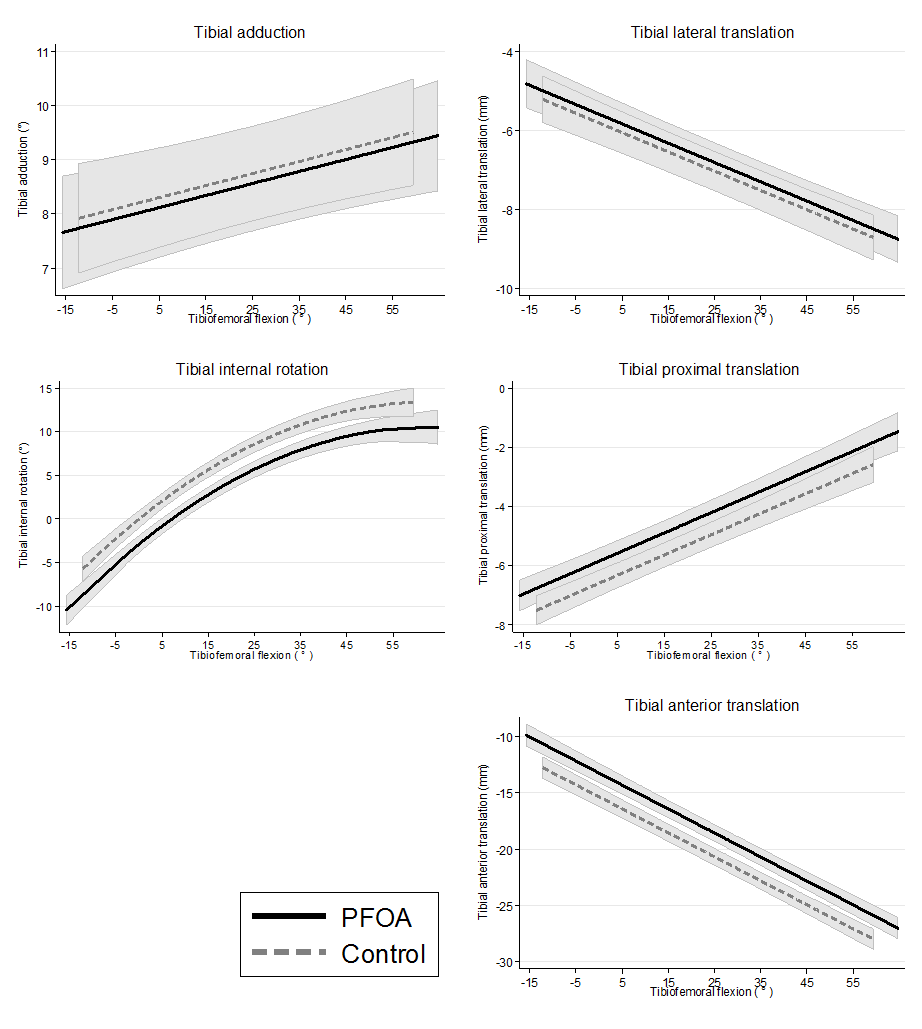


FIGURE S2. Fitted 3D tibial alignment in PF OA cases (solid black) and controls (dashed grey). Gray shading represents one standard deviation above and below the group means.

**TABLE S2. Between-group difference in alignment of matched pairs**

(PFOA minus control [CON]) in standing across four angles, with Cohen’s d.

|  | Two-legged, 0$\boldsymbol{^{\circ}}$ | | Two-legged, 15$\boldsymbol{^{\circ}}$ | | Two-legged, 30$\boldsymbol{^{\circ}}$ | | Two-legged, 45$\boldsymbol{^{\circ}}$ | |
| --- | --- | --- | --- | --- | --- | --- | --- | --- |
| Tibiofemoral joint | **PFOA - CON** | **d** | **PFOA - CON** | **d** | **PFOA - CONT** | **d** | **PFOA - CONT** | **d** |

| Adduction | -0.3 (4.5) | -0.1 | -0.8 (4.2) | -0.2 | -0.4 (4.0) | -0.1 | 1.0 (4.3) | 0.1 |
| --- | --- | --- | --- | --- | --- | --- | --- | --- |
| Internal rotation | -2.0 (7.0) | -0.3 | -2.2 (4.3) | **-0.5** | -2.9 (5.3) | **-0.5** | -2.7 (4.0) | **-0.7** |
| Proximal translation | 0.7 (2.1) | 0.3 | 1.1 (2.1) | **0.5** | 0.5 (2.4) | 0.2 | 1.3 (3.6) | 0.4 |
| Lateral translation | 0.0 (2.5) | 0.0 | 0.3 (2.9) | 0.1 | -0.0 (2.6) | 0.0 | 0.3 (3.2) | 0.1 |
| Anterior translation | 1.8 (3.7) | **0.5** | 1.2 (5.3) | 0.2 | 2.3 (5.2) | 0.4 | 1.9 (6.6) | 0.3 |

**bold indicates Cohen’s |d| ≥ 0.5*
